# *Theileria* parasites subvert E2F signaling to stimulate leukocyte proliferation

**DOI:** 10.1038/s41598-020-60939-x

**Published:** 2020-03-04

**Authors:** Kyle Tretina, Malak Haidar, Sally A. Madsen-Bouterse, Takaya Sakura, Sara Mfarrej, Lindsay Fry, Marie Chaussepied, Arnab Pain, Donald P. Knowles, Vishvanath M. Nene, Doron Ginsberg, Claudia A. Daubenberger, Richard P. Bishop, Gordon Langsley, Joana C. Silva

**Affiliations:** 1 Institute for Genome Sciences, University of Maryland School of Medicine, Baltimore, MD 21201 USA; 2 Program in Molecular Microbiology and Immunology, University of Maryland School of Medicine, Baltimore, MD 21201 USA; 3 Laboratoire de Biologie Comparative des Apicomplexes, Faculté de Médicine, Université Paris Descartes, Sorbonne Paris Cité, France; 4 Inserm U1016, Cnrs UMR8104, Cochin Institute, Paris, 75014 France; 5 Department of Veterinary Microbiology and Pathology, Washington State University, Pullman, WA 99164-7040 USA; 6 Pathogen Genomics Laboratory, Biological and Environmental Sciences and Engineering (BESE) Division, King Abdullah University of Science and Technology (KAUST), Thuwal, 23955-6900 Kingdom of Saudi Arabia; 7 Animal Disease Research Unit, Agricultural Research Service, USDA, Pullman, WA 99164-7030 USA; 8 Weizmann Institute of Science, Molecular Cell Biology Department, PO Box 26, Rehovot, 76100 Israel; 9 International Livestock Research Institute, Nairobi, 00100 Kenya; 10 The Mina and Everard Goodman Faculty of Life Sciences Bar-Ilan University, Ramat-Gan, 52900 Israel; 11 Swiss Tropical and Public Health Institute, Basel, Switzerland; 12 University of Basel, Basel, Switzerland; 13 Department of Microbiology and Immunology, University of Maryland School of Medicine, Baltimore, MD 21201 USA

**Keywords:** Data mining, Parasite host response

## Abstract

Intracellular pathogens have evolved intricate mechanisms to subvert host cell signaling pathways and ensure their own propagation. A lineage of the protozoan parasite genus *Theileria* infects bovine leukocytes and induces their uncontrolled proliferation causing a leukemia-like disease. Given the importance of E2F transcription factors in mammalian cell cycle regulation, we investigated the role of E2F signaling in *Theileria*-induced host cell proliferation. Using comparative genomics and surface plasmon resonance, we identified parasite-derived peptides that have the sequence-specific ability to increase E2F signaling by binding E2F negative regulator Retinoblastoma-1 (RB). Using these peptides as a tool to probe host E2F signaling, we show that the disruption of RB complexes *ex vivo* leads to activation of E2F-driven transcription and increased leukocyte proliferation in an infection-dependent manner. This result is consistent with existing models and, together, they support a critical role of E2F signaling for *Theileria*-induced host cell proliferation, and its potential direct manipulation by one or more parasite proteins.

## Introduction

*Theileria parva* and *Theileria annulata* are tick-transmitted, protozoan parasites of cattle that cause hundreds of millions of dollars of economic losses every year in large parts of southern Europe, Africa, and southern Asia. These pathogens are notable among protozoan parasites in the ability of the schizont life-cycle stage to induce cancer-like phenotypes in host leukocytes. Induction of proliferation and dissemination of infected host cells are believed to be important aspects of *Theileria-*induced pathogenesis^1,2^. Despite a strong host immune response to the parasites^3^, infection can result in the death of a high proportion of infected animals^4^. While there has been considerable research into host signaling pathways affected during infection, so far only one parasite molecule has been shown to be involved in *Theileria*-induced transformation of host leukocytes^1^. This protein, a prolyl isomerase secreted by *T. parva* and *T. annulata* into the leukocyte cytosol, modifies host oncogenic proteins critical for host cell transformation^5^. Notably, the host ortholog of this parasite protein, PIN1^6^, as well as several other host genes that are critical for transformation^7^ are dependent on the activation of the host’s E2F cell-cycle regulators and transcription factors. E2F proteins have been previously postulated to be activated during *Theileria* infection through IL-2 and PI3K-dependent PKB/AKT signaling^7^. More recently, E2F-1, E2F2, and E2F3 were shown to be highly expressed, but slightly down-regulated at the transcript level in *Theileria-*transformed cells compared to uninfected controls^8^; however, the functional role of E2F signaling in *Theileria*-induced host cell proliferation has not yet been thoroughly characterized.

Here, we leveraged a comparative genomics approach to predict parasite protein sequences that are potentially involved in the *Theileria*-induced transformation of host leukocytes. We discovered a peptide derived from a parasite protein that has the sequence-specific and infection-specific ability to increase host E2F signaling by binding to the Retinoblastoma-1 (RB), a negative regulator of E2F signaling, and thereby contributing to host cell hyper-proliferation. Hence, cumulative evidence to date demonstrates the critical role of the RB/E2F signaling axis in *Theileria* host cell proliferation, and suggests a mechanism by which one or more parasite proteins could manipulate E2F signaling.

## Results

**Figure 1:**
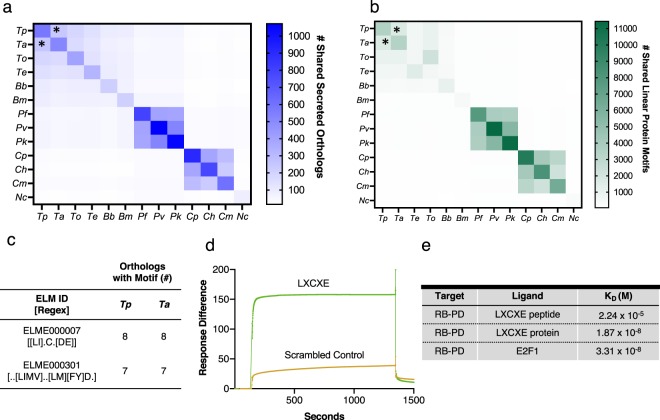
Comparative genomics identified 15 *Theileria* parasite proteins with the potential to alter host E2F signaling, one of which can bind Retinoblastoma-1 pocket domain. (**a**) A heatmap of orthologous proteins that are shared by each pair of apicomplexan parasites used in this study. (**b**) A heatmap of short eukaryotic linear motifs in secreted proteins that are shared by pairs of apicomplexan parasites used in this study. (**c**) 15 *Theileria* secreted proteins have short linear motifs that are present in the Eukaryotic Linear Motif (ELM) database and are predicted to interact with host Retinoblastoma-1 (RB). (**d**) A kinetic surface plasmon resonance binding curve (sensorgram) showing that peptides derived from one of the proteins in (**c**) binds RB in a sequence-specific manner, as shown by surface plasmon resonance. (**e**) Listed are estimated dissociation constants for the LXCXE peptide (same as (**d**)), a full-length *Theileria* protein containing that peptide (TpMuguga02_00667; *Tp*GcpE), as well as for host E2F1, binding to the RB pocket domain.

We developed a bioinformatics pipeline to perform a comprehensive prediction of potential host-transforming, short linear motifs in *Theileria* proteins. This pipeline was based on the rationale that such peptide motifs have all of the following attributes: (*i*) are in proteins predicted to be exposed to the host (i.e. secreted), (*ii*) are present in host-transforming *Theileria* species (*T. parva* and *T. annulata*), and (*iii*) are found in proteins that are expressed in the schizont life-cycle stage based on RNA-seq data. In order to reduce false positives, we also imposed a strict criterion that the protein motif be absent in the protein sequences of orthologs found in other apicomplexan genomes, including non-transforming *Theileria* (*T. orientalis* and *T. equi*). While only 400 protein orthologs were unique to *T. parva* and *T. annulata*, a total of 109 protein motifs, present in these 400 proteins, satisfied the required criteria (Fig. 1a,b; Supplementary Dataset). This list is critically dependent on a recently generated *T. parva* genome annotation, since the coding sequences of 101 of these 400 genes were altered in the new annotation (Tretina *et al*., submitted)^9^.

Interestingly, we identified 15 *T. parva* proteins that contain a short linear motif predicted to interact with the well-studied host tumor suppressor Retinoblastoma-1 protein (RB) (Fig. 1c, Supplementary Table 1). Along with p107 and p130, RB is a pocket family protein that plays a critical role in mammalian cell cycle regulation by inhibiting E2F transcription factor activity, an important regulator of cell proliferation and survival^10^. The central RB pocket domain is highly conserved between humans and bovids (Supplementary Fig. 1) and has two significant binding sites: a) the interface of the A and B cyclin folds, which binds to the E2F transactivation domain LXXLFD motif, and (b) a three-helix cleft of the B cyclin fold, which binds to chromatin-modifying proteins containing an LXCXE motif, such as histone deacetylase 1 (HDAC1)^10^. Out of our 15 predicted *T. parva* RB-binding proteins, eight had the LXCXE motif, and seven had the LXXLFD motif (Supplementary Table 1).

Since the proliferation of *T. parva*-infected host cells is thought to be critical for the pathogenesis of infection, we investigated whether synthesized peptides, each 9 to 19 amino acids long and containing these 15 *T. parva* motifs, could bind to RB (Supplementary Table 1). Surface plasmon resonance (SPR) was used to screen these peptides for specific RB1 pocket domain-binding activity. Recombinant, purified RB pocket domain was coupled to the surface of the sensor chip, and purified peptides were introduced at various concentrations to quantify kinetics if response curves indicated a detectable protein-protein interaction. Sequence-scrambled peptides were used as negative controls to exclude false-positive signals. This approach led to the discovery of two peptides that bind RB in a sequence-specific fashion, one with an LXCXE motif (in TpMuguga_02g00667, *Tp*GcpE, Fig. 1d,e), and one with an LXXLFD motif (in TpMuguga_02g02355). SPR kinetics assays revealed that full-length, recombinant *Tp*GcpE protein also binds the RB pocket domain with nanomolar affinity (18.7 nM) (Fig. 1e).

**Figure 2:**
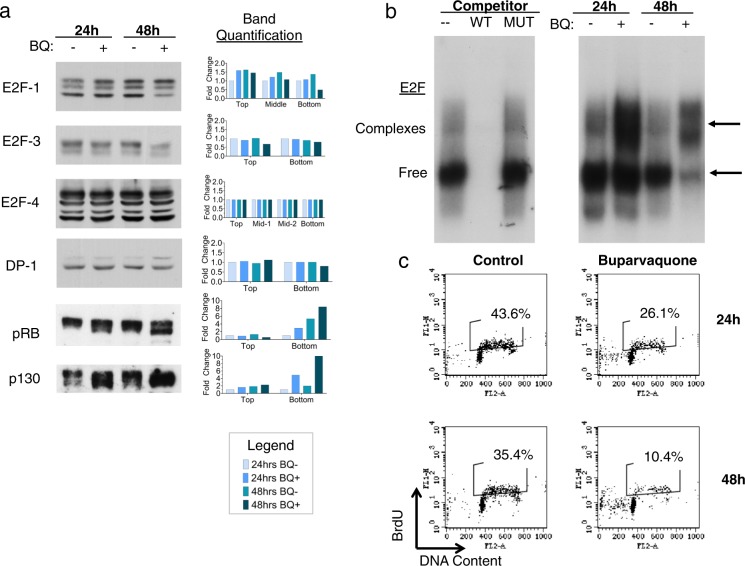
RB/E2F signaling is activated by *Theileria* parasites. (**a**) Western blot analysis (left) of E2F-1, E2F-3 E2F-4, DP-1, pRB, and p130 expression in TpMD409B.2 cells, either left untreated (−), or treated (+) for 24 h and 48 h with buparvaquone (BQ), including band intensity quantification (right). Shown are representatives of three independent experiments. (**b**) E2F DNA binding activity in TpMD409B.2 cells was assayed by EMSA using gP32- labeled double-stranded oligonucleotide spanning E2F binding sites from the *dhfr* gene promoter. Left panel, nuclear extracts from TpMD409B.2 cells were incubated with γP32-labeled probe in absence or presence of a 100 fold molar excess of unlabeled wild type (WT), or mutated (MUT) double-stranded oligonucleotide. Binding reactions were resolved by non-denaturing polyacrylamide gel, which was processed afterwards for autoradiography. Right panel, EMSA analysis of E2F DNA binding activity in nuclear extracts from untreated, proliferating TpMD409B.2 cells (−) and TpMD409B.2 cells treated for 24 and 48 h with the parasiticidal drug buparvaquone (+). Arrows indicate E2F in complex with RB family proteins (Complexes) or free E2F protein (Free). (**c**) Flow cytometric analysis of BrdU incorporation and DNA content in TpMD409B.2 cells, either left untreated (control), or treated with buparvaquone for 24 h and 48 h. Shown is a representative of three independent experiments.

The discovery of these peptides led us to investigate the particular RB/E2F signaling components in *Theileria*-infected cells. Eight E2F transcription factor genes have been identified in mammals, which can be divided into two groups: activators (E2F-1, E2F-2, E2F-3a), which can bind RB, and repressors (e.g. E2F-3b, E2F-4, E2F-5), which can bind RB, p107 and p130. The balance between the activator and repressor E2Fs seems to be primarily controlled by the phosphorylation status of the associated pocket proteins to which they bind. E2F interactions with the Dimerization Partner (DP) ligands are also important for regulating E2F transcription factor activity^11^. We used Western blot to compare the levels of RB family, E2F, and DP-1 protein between *T. parva*-transformed cells that were left untreated with those treated with buparvaquone to kill the parasite and induce an arrest in host cell proliferation. By comparing expression levels between the buparvaquone-treated and untreated groups, we can determine parasite-dependent changes in the expression levels of these proteins. We found that the *T. parva*-transformed B cell line TpMD409.B2 expresses RB, p130, E2F-1, E2F-3, E2F-4, DP-1 (Fig. 2a). It is noteworthy that phosphorylated RB (pRB) and p130 migrate faster in parasite-killed TpMD409.B2 cells than in untreated cells (Fig. 2a). These faster migrating bands likely correspond to hypophosphorylated, proliferation-suppressive forms of RB and p130, at 24 h and 48 h after treatment with buparvaquone. Interestingly, after 48 h of treatment, but not 24 h, E2F-1 and E2F-3 protein levels decrease, a slower-migrating DP-1 band increases, but E2F-4 levels remain unaltered (Fig. 2a). This indicates that, in the presence of live intracellular *T. parva* parasites, host cells express higher E2F-1 and E2F-3 levels, DP-1 has decreased post-translational modifications, and RB and p130 are hyper-phosphorylated. Furthermore, the binding of E2F to DNA appears to increase in the presence of live parasites, since treatment with the parasiticidal drug buparvaquone over 24 h and 48 h is accompanied by an overall decrease in E2F DNA binding activity, as well as by an increase in complexes migrating at slow and intermediate speed bound to an E2F-dependent promoter, which likely represent E2F/RB repressor complexes (Fig. 2b). Proliferation was also confirmed to be dependent on the parasite by BrdU incorporation of untreated and buparvaquone-treated *T. parva* infected cells (Fig. 2c), as has been shown previously^8^.

**Figure 3:**
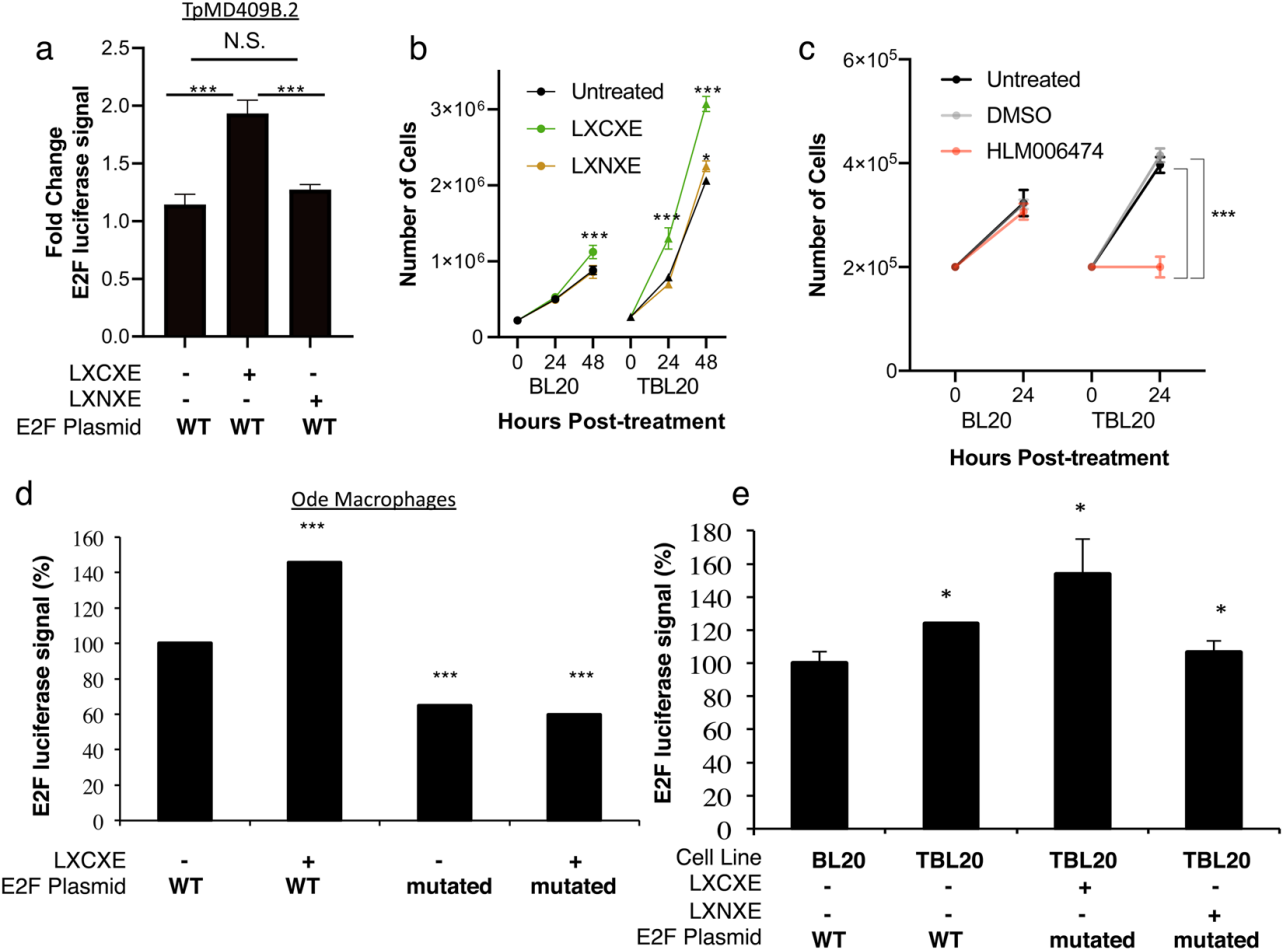
Sequence-specific disruption of RB/E2F signaling with cell permeating peptides reveals the active role of E2F signaling during *Theileria* infection. (**a**) TpMD409B.2 cells were transfected with E2F-luc (WT = wild-type E2F binding construct), or mutated E2F-luc constructs (labeled ‘mutated’) together with a CMV-lacZ plasmid to normalize for transfection efficiency. Luciferase and β-galactosidase activities were measured 24 hrs after transfection. E2F-luc relative luciferase activity normalized to mE2F-luc. Shown is the average of 3 independent experiments with standard deviation. BL20 or TBL20 cells were treated with (**b**) 1μM of penetrating peptides (VKKKKIKREIKIYIEEVFTPLV**L**K**C**K**E**LK-K(FITC)) containing the *Tp*GcpE LXCXE motif, or an LXNXE control (VKKKKIKREIKIYIEEVFTPLV**L**K**N**K**E**LK-K(FITC)) or (**c**) E2F inhibitor or DMSO control, and then counted by hemacytometer after the indicated timepoints. Shown is the average of 3 independent experiments with standard deviation. Then, either (**d**) virulent Ode *T. annulata*-transformed macrophages, or (**e**) BL20/TBL20 *T. annulata*-transformed cells were transfected with E2F-luc plasmid (or mutated E2F-luc as in (**c**)) and incubated for 24 hrs at 37 **°**C. Then, cells were treated with penetrating peptides as in (**b**) for 2 hrs and luciferase activity was quantified. Data shown are representative of three independent experiments done with biological duplicates, with mean + standard deviation (*P-value < 0.05 compared to untreated; ^‡^*P*-value < 0.05 compared to its respective BL20 control).

We also found that *T. parva*-transformed B cells, TpMD409.B2 cells, contain significant levels of E2F transcriptional activity, since cells transfected with an E2F-driven luciferase reporter plasmid (E2F-luc) exhibited a 200% increase in luciferase activity compared to cells transfected with a plasmid containing a mutated E2F binding site (mutated) (Fig. 3d). Since no system for genetic manipulation is available yet for intracellular *Theileria*, we instead incubated *T. parva-* and *T. annulata-*infected bovine leukocytes (transfected with a luciferase reporter regulated from an E2F promoter) with synthesized peptides containing the RB-binding LXCXE motif, or a mutated LXNXE negative control, linked to a cell-penetrating peptide. If the LXCXE-containing peptide can regulate E2F activity in a sequence-specific manner, we would expect it to increase E2F activity, and that this effect would be reduced or abrogated in the mutated LXNXE peptide-treated group. As expected, the LXCXE peptide increased E2F activity in *T. parva*-infected B cells, as well as in *T. annulata*-infected B cells and macrophages. In contrast, the mutated LXNXE peptide did not show the same reporter activity as the LXCXE peptide (Fig. 3b,d,e), providing direct functional evidence that the parasite-derived LXCXE motif can induce E2F activity in *Theileria*-infected leukocytes.

Since the LXCXE peptide can induce E2F activity, we investigated whether it could, on its own, increase bovine leukocyte proliferation. To extend our observations to *T. annulata*-infected leukocytes, we incubated *T. annulata*-infected B cells with WT or mutated LXNXE peptides and counted cell numbers at 24 h and 48 h. The addition of these peptides had no effect on cell viability in any of the experimental groups (data not shown). Importantly, only the LXCXE peptide induced a significant increase in proliferation of *T. annulata*-infected B cells as well as (to a much lesser extent) of uninfected, but immortalized controls (Fig. 3b). Interestingly, an E2F inhibitor (HLM006474) specifically reduced the proliferation of *T. annulata*-infected TBL20 cells and not uninfected BL20 cells (Fig. 3c), an effect that was not dependent on cell death (data not shown). These data demonstrate that RB/E2F interactions significantly regulate *Theileria*-driven host leukocyte proliferation.

**Figure 4:**
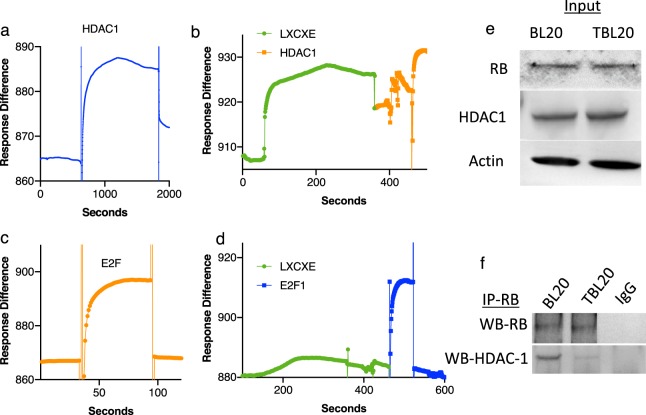
An LXCXE motif-containing protein disrupts RB interactions with negative epigenetic regulators of E2F signaling, but not RB-E2F interactions. (**a**) A kinetic surface plasmon resonance binding curve (sensorgram) of full-length HDAC1 to the RB pocket domain is shown. (**b**) Kinetic binding curve of full-length HDAC1 in competition with full-length TpMuguga02_00667 (*Tp*GcpE). (**c**) A sensorgram of full-length E2F1 to the RB pocket domain is shown. (**d**) Kinetic binding curve of full-length E2F1 in competition with full-length TpMuguga02_00667. (**e**) Western blot analysis of RB and HDAC1 in BL20 and TBL20 cells. (**f**) Co-immunoprecipitation of RB and HDAC1 in RB immunoprecipitates. Shown is a representative of three independent experiments.

In order to map the site of the interaction of the LXCXE motif with the RB pocket domain, we used SPR to test the ability of full-length *Tp*GcpE protein to compete with two proteins that have known, well-characterized interactions with the RB pocket domain, E2F1 and HDAC1. The RB pocket domain was coupled to the surface of the sensor chip, and purified, recombinant, full-length *Tp*GcpE was introduced into the flow channel to bind the RB pocket domain to saturation; then either purified, recombinant E2F-1 or HDAC1 was added to the channel and the response curve recorded to determine if they competed with*Tp*GcpE binding. We found that E2F-1 does not compete with full-length *Tp*GcpE for binding to the RB pocket domain (Fig. 4a,b). However, *Tp*GcpE does compete with HDAC1 for RB (Fig. 4c,d), despite a > 4.5-fold higher concentration of the latter, suggesting that the full-length *Tp*GcpE can compete with endogenous chromatin-modifying enzymes like HDAC1 for binding to the LXCXE-binding cleft of the RB pocket domain. We also found that RB interacts with HDAC1 in uninfected cells, and to a lesser extent, in infected cells, as detected by co-immunoprecipitation (Fig. 4b,e,f). This could be partially explained by competition of HDAC1 with LXCXE-containing proteins, as total RB and HDAC1 levels do not seem to be altered by the presence of *T. parva* (Fig. 4e).

## Discussion

RB binds and represses E2F transcription factor family proteins in non-proliferating cells, in part by recruiting LXCXE motif-containing chromatin modifiers to E2F-dependent promoters. Mitogenic signals lead to an increase in cellular cyclin-dependent kinase activity, which phosphorylates RB and thereby inactivates RB-dependent E2F repression by dissociating the RB-E2F complex. Interestingly, the RB LXCXE-binding cleft is also critical for repressing transcription from the cyclin E and cyclin A promoters. Since cyclin E/A-CDK2 complexes can phosphorylate and inactivate RB, this provides an important positive feedback signal for proliferation^12^. Free E2F proteins form heterodimers with E2F dimerization partner proteins DP-1 or DP-2 to form a functional transcription factor that regulates cell proliferation and survival^10,13^.

While full-length *Tp*GcpE was measured to have very strong binding to host RB by SPR, the LXCXE-containing peptide derived from *Tp*GcpE bound with lower affinity than the full-length protein (Fig. 1). This is indicative of the growing consensus that although the LXCXE motif can be used to identify potential RB-interaction partners, structural studies with both pathogen-derived and mammalian host-derived proteins indicate that there are other important determinants of high-affinity interactions with the pocket cleft^14^. For example, out of the eight LXCXE motif-containing peptides testing in this study, only one bound RB. This is supported by other studies finding that several proteins that interact with this region of RB do not contain an obvious LXCXE motif^15,16^, suggesting that RB interactions in the LXCXE-binding cleft are more complicated than currently understood from structural studies.

While *Tp*GcpE was measured to have very strong binding to host RB by SPR (Fig. 1), immunofluorescence imaging with a polyclonal antibody (Supplementary Fig. 2) found very little signal outside of the parasite co-localizing with host RB. There can be a few alternative, non-competing explanations for our limited ability to detect GcpE in the host cell cytosol: (a) the signal peptide in GcpE is also predicted to contain a motif targeting it to the apicoplast (a chloroplast-like organelle specific of the Apicomplexa), suggesting that only small amounts of GcpE remain in the host cell cytosol, and at levels below antibody detection; (b) during host cell division the multinucleated macroschizont is partitioned into new daughter host cells and enough GcpE gets released into the cytosol to sustain E2F activation; or (c) antagonism of the RB/HDAC1 binding by *Theileria* parasites results from competition with more than one parasite-derived motif, since we identified 15 *T. parva* proteins predicted to interact with RB, where eight had the LXCXE motif and seven had the LXXLFD motif. Moreover, SPR demonstrated that the LXCXE motif (in TpMuguga_02g00667, *Tp*GcpE), and the LXXLFD motif (in TpMuguga_02g02355) bound the RB pocket domain with nanomolar affinity. Examining all 15 proteins for their presence in the host cell cytosol and eventual interaction with RB will be necessary to test hypothesis (c). It should also be noted that the parasite marker used for staining the schizont (p104), is secreted by the parasite and then subsequently localizes to the parasite surface, suggesting that this staining may illuminate more than just the parasite intracellular compartment. However, there is some weak GcpE staining outside of the p104-stained regions, indicating that some GcpE may localize to the host nucleus.

Our demonstration that penetrating peptides linked to the LXCXE motif in GcpE can block RB-HDAC1 binding, activate E2F-driven transcription and promote infected leukocyte proliferation, argues that targeting the RB/E2F signaling pathway could lead to novel chemotherapeutics against *T. annulata-*induced pathogenesis. For example, drugs have recently been developed to prevent binding of LXCXE motif-containing HPV E7 protein to host RB and are selectively cytopathic to HPV-infected cells^17^. In fact, the E2F inhibitor HLM0006474 was able to specifically inhibit the proliferation of *T. annulata*-infected, but not uninfected bovine B cell lines (Fig. 3c), indicating that E2F signaling could be a viable target of chemotherapeutic intervention for this infection. Since *T. parva* and *T. annulata* bind to their host’s mitotic spindle and divide in synchrony with the host leukocytes^18^, host proliferation is intricately linked to parasite proliferation. This unique mechanism of parasite proliferation also provides the parasite with immune protection, as it avoids parasite egress from the host cell and exposure to the host immune response. We have established that uncontrolled proliferation of *Theileria-*transformed leukocytes can be achieved through modulation of RB/E2F signaling, and that *Tp*GcpE has the potential to alter those interactions. Conclusive demonstration that specific *Theileria* proteins manipulate host RB/E2F signaling *in vivo* awaits future studies.

## Methods

### Bioinformatic analyses

#### Comparative genomics

Jaccard-filtered eukaryotic clusters of orthologous genes (KOGs) were generated as described previously^19^, using the following genomes from EuPathDB (August 25^th^, 2014)^20^: *Babesia bovis* T2Bo, *Cryptosporidium hominis* TU502, *Cryptosporidium muris* RN66, *Cryptosporidium parvu*, Iowa II, *Neospora caninnum* Liverpool, *Plasmodium falciparum* 3D7, *Plasmodium knowlesi* strain H, *Plasmodium vivax* Sal-1, *Theileria annulata* strain Ankara, *Theileria equi* strain WA, and *Theileria orientalis* strain Shintoku. Also used for creating the KOGs were the updated *Theileria parva* strain Muguga annotation and an updated *Babesia microti* RI annotation recently completed by our group^21^. Localization predictions were made with TargetP^22^, transmembrane domain predictions with TMHMM^23^, GPI predictions were made with GPI-SOM^24^, all using the default parameters. The entire ELM database was downloaded from http://elm.eu.org/ and custom scripts were used to search the proteome of each Apicomplexan in the Sybil database using regular expressions in Python and a *p*-value cutoff of 0.001.

### Surface plasmon resonance experiments

#### Immobilization of human RB1 protein to CM5 chip

*Surface preparation*. Binding reactions were done in HBS-EP buffer from Biacore (Biacore Inc., New Jersey), containing 10 mM Hepes, 150 mM NaCl, 3 mM EDTA, and 0.05% (v/v) surfactant p20, pH 7.4, filtered (0.2 µM) and degassed before use. Protein human Retinoblastoma-1 (LDBiopharma HTF-1035) was created on the surface of a BIAcore CM5 sensor chips by direct immobilization. The carboxymethyl-dextran surface of the chip (flow cell 2, 3 and 4) were activated with a 35 µl injection of a mixture of 0.1 M NHS and 0.1 M EDC in water. An aliquot of 100 µl of 10 µg/ml dilution of human Retinoblastoma-1 protein in 10 mM sodium acetate, pH 4.5 was injected into flow cell-2 of CM5 chip to get the levels of 2000 resonance units (RU) for the immobilization. The remaining NHS-ester active sites in the dextran surface flow cell-2 was blocked with 35 µl of 1 M ethanolamine, pH 8.2 and washed at a high flow rate, 100 µl per minute, with one pulse of 100 µl of HBS-P buffer, pH 7.4. The flow cell-1 of same CM5 chip was used as reference (activated with a 35 µl injection of a mixture of 0.1 M NHS and 0.1 M EDC in water and blocked with 35 µl of 1 M ethanolamine, pH 8.2).

*Kinetics analysis of binding*. To minimize mass transport effects, the binding analyses were performed at flow rate of 30 µl per minute at 25 °C. The analytes (60 µl each, 0–25 µM in HBS-EP buffer with 0.05% P20) were injected into flow cell and the association of analyte and ligand were recorded respectively by surface plasmon resonance (SPR) with a Biacore T200 (Biacore, Inc., New Jersey). After this, the surface was washed with buffer for 180 seconds to follow the dissociation of analyte-ligand complexes. The signal from the blank channel (flow cell-1) was subtracted from the channel containing human Retinoblastoma-1 protein. The binding was removed by injecting 100 µl of HBS-P, pH 7.4.

*Data analysis*. Sensorgrams of the interaction generated by the instrument were analyzed using the software BIAeval 3.2 (Biacore Inc., New Jersey). The reference surface data were subtracted from the reaction surface data to eliminate refractive-index changes of the solution, injection noise and non-specific binding to the blank surface. A blank injection with buffer alone was subtracted from the resulting reaction surface data. Data was globally fitted to the steady state affinity.

### Cell culture and transient transfections

The TpMD409.B2 cell line is a *T. parva Muguga*-infected B-cell clone (B2) whose establishment, phenotypic characteristics and culture conditions have been described^25–27^. BL20 cells are a retrovirus-transformed B cell line isolated from an infected cow. To eliminate the parasite, cells were treated for the indicated time with buparvaquone (50 ng/ml, 1 mg/ml stock in ethanol; Sigma). To inhibit E2F activity, cells were treated for the indicated time with HLM006474 (10 μM in DMSO, Sigma), or DMSO (10μM; Sigma) as a control. The *T. annulata*-infected cell lines BL20 (B cell), as well as Ode (macrophage) cell lines are well described and characterized.

### Reporter gene assays

TpMD409.B2 cells were co-transfected with 20 μg (E2F-luc, mE2F-luc), or 10 μg (cyclin E-luc) reporter construct, 20 μg of expression vector and 5 μg of pCMV-lacZ, where indicated. Luciferase and β-galactosidase activities were measured 24 h after transfection according to previously published procedures^25^. TpMD409.B2 cells were also co-transfected with pCMV-HA-DP-1 D103–126 and *c-myc*-luciferase as previously described^28^.

### Flow cytometric analysis of BrdU incorporation

TpMD409.B2 cells were transfected as described for cell cycle analysis except that H2B-EGFP expression vector (10 μg) was used instead of phGFP-S65T. 48 h after transfection, cells were pulse labeled for 1 h with BrdU (5-bromo-2′-deoxyuridine) (10 μg/ml) then harvested and fixed in 1% paraformaldehyde, 0.01% Tween 20 for 48 h to 72 h at 4 °C. BrdU staining was performed using phycoerythrin-labeled anti-BrdU antibody (Pharmingen) according to the previously published DNase I procedure^29^. A minimum of 2,000 events in each whole population and GFP positive fraction were collected on a FACSCAN flow cytometer and analyzed with CellQuest.

Alternatively, untreated and buparvaquone treated TpMD409.B2 cells, pulse labeled for 30 min with BrdU, were fixed in 70% ethanol overnight at −20 °C. BrdU staining was performed using fluorescein isothiocyanate-labeled anti BrdU antibody (Boehringer Mannheim) following the acid denaturation procedure. DNA was counter-stained for 30 min at room temperature with propidium iodide (5 μg/ml) in PBS containing RNase (200 μg/ml). 10,000 events were analyzed on a FACSCAN.

### Cells extracts, antibody and Western Blot analysis

TpMD409.B2 cells were washed twice in ice-cold PBS then lysed in ice-cold lysis buffer consisting of 10 mM HEPES pH 8, 150 mM NaCl, 1% NP40, 0.5% sodium deoxycholate, 0.1% SDS to which proteases (Complete, Roche) and phosphatases (5 mM Na_3_VO_4_, 50 mM NaF) inhibitors were added. Cell debris was pelleted by centrifugation at 13,000xg for 10 min at 4 °C and proteins were quantified by the Bradford method. Equal amounts of proteins were resolved by SDS-polyacrylamide gel and electro-transferred to nitrocellulose. The following antibodies were used in Western Blot analysis: anti-RB (1:1000) and anti-DP-1 (TFD10) (1:500) from Pharmingen, anti-p130 (C-20) (1:400), anti-E2F-1 (KH95) (1:500), anti-E2F-3 (C1–18) (1:1000) and anti-E2F-4 (C-108) (1:300) from Santa Cruz Biotechnology. For the generation of antibodies, recombinant *Ta*GcpE protein was generated using a pGEX6P-1 plasmid that places a GST-tag at the N-terminal end of the expressed recombinant protein. Fragments encoding both the N-terminus (17–412 aa) and C-terminus (413–808 aa) of *Ta*GcpE were amplified from cDNA of *T. annulata* and cloned into the pGEX6p-1 plasmid. The 2 recombinant *Ta*GcpE proteins were expressed in BL21 CodonPlus (DE3)-RIPL and cell pellets were suspended in PBS supplemented with protease inhibitor, 1 mM EDTA, 1 mg/ml lysozyme, then sonicated and centrifuge 10,000 rpm for 1 h at 4 °C. Cell lysates were incubated with glutathione sepharose beads, and recombinant proteins were eluted by elution buffer (10 mM glutathione, 50 mM Tris-HCl pH 8.0). Since the N-terminal half of *Ta*GcpE was not soluble, sera were raised against the soluble C-terminal half of TaGcpE by immunizing two rats according to “the Speedy 28-day protocol” (Eurogentec).

### Immunofluorescence assays

5 × 10^4^ cells were plated on glass coverslips coated with poly-L-lysine. Cells were fixed with 4% paraformaldehyde in PBS for 10 min, and permeabilized using 0.5% Triton X-100. After a 30 min treatment with blocking solution (1% BSA and PBST buffer: 0.1% Tween20), cells were stained for 2 h with the primary antibodies (anti-RB: Santa Cruz Biotechnology #sc-73598, anti-1C12, and a rat anti-GcpE antibody. After three washes with PBS, cells were incubated with the secondary antibody (Alexa fluor 488 donkey anti-rat IgG #A21208 and Alexa fluor 594 goat anti-mouse IgG #A11005) for 45 min in the dark. After three additional washes, slides were stained with 4–6-diamidino-2-phenylindole, diluted 1/1000 (Sigma Aldrich) for 5 min to visualize the nuclei. Labelled preparations were mounted in Dako and analysed by inverted microscopy (Leica DMI6000s). Acquisitions were made with the Metamorphous software.

### Electrophoretic mobility shift assay (EMSA)

Nuclear extracts were prepared as described previously^30^. 5 μg of nuclear extracts were incubated for 30 min at room temperature with gP32 labeled double stranded oligonucleotide in binding buffer (5 mM HEPES Ph 8, 0.5 mM EDTA, 5% glycerol, 0.5 mM DTT, 0.5 M PMSF, 0.5 mM MgCl2, 0.005% NP40, 1 mg/ml BSA, sonicated salmon sperm DNA 100 μg/ml). The complexes were resolved on a non-denaturing 4% polyacrylamide gel. The following oligonucleotides were used:

E2F – WT: 5′ CTAGTGCAATTTCGCGCCAAACTTG 3′

E2F – MUT: 5′ CTAGTGCAATTGCTCGACCAACTTG 3′

### Peptide synthesis

The amino acid sequence of RB-1 based on the parasite sequence (VKKKKIKREIKIYIEEVFTPLVLKCKELK) was synthesized. Cysteine in wild type RB1 was replaced by an Asparagine (**N**; in bold VKKKKIKREIKIYIEEVFTPLVLK**N**KELK). The penetrating peptide to facilitate cell entry is underlined. 3 different concentrations of peptides were used for 2 h to determine the minimum effective concentration (1 μM) with minimum toxic effect, which was used to perform each experiment as indicated.

### Statistical analysis

Student’s two-tailed T-tests were performed for cell culturing experiments with at least *N* = 3 for each experiment. Sample size was chosen based on literature references for similar experiments in other systems. The values for *N*, *P*, and the specific statistical test performed for each experiment are included in the appropriate figure legends or main text. In each figure or text, the following symbols represent respective p-value ranges: *0.05 > *p* ≥ 0.01; **0.01 > *p* ≥ 0.001, ***p < 0.001. Differences were considered significant if *p* < 0.05.

## Supplementary information

Supplemental_Figs-Tables.

Suppl-Dataset-1.
