# Supplementary material for: Theileria parasites subvert E2F signaling to stimulate leukocyte proliferation
Source: Sci Rep. 2020 Mar 4;10:3982. doi: 10.1038/s41598-020-60939-x (PMC7055300; doi:10.1038/s41598-020-60939-x)
Supplement: Supplementary file 1 — Supplemental_Figs-Tables. [file 41598_2020_60939_MOESM1_ESM.docx]

***Theileria* parasites subvert E2F signaling to stimulate leukocyte proliferation**

**Authors:**

Kyle Tretina^1,2^, Malak Haidar^3,4^, Sally A. Madsen-Bouterse^5^, Takaya Sakura^3,4^, Sara Mfarrej^6^, Lindsay Fry^5^, Marie Chaussepied^3,4,7^, Arnab Pain^6^, Donald P. Knowles^5,8^, Vishvanath M. Nene^9^, Doron Ginsberg^6,10^, Claudia A. Daubenberger^11,12^, Richard P. Bishop^5^, Gordon Langsley^3,4^, Joana C. Silva^1,13*^

**Supplemental Information**

**Supplementary Figures**


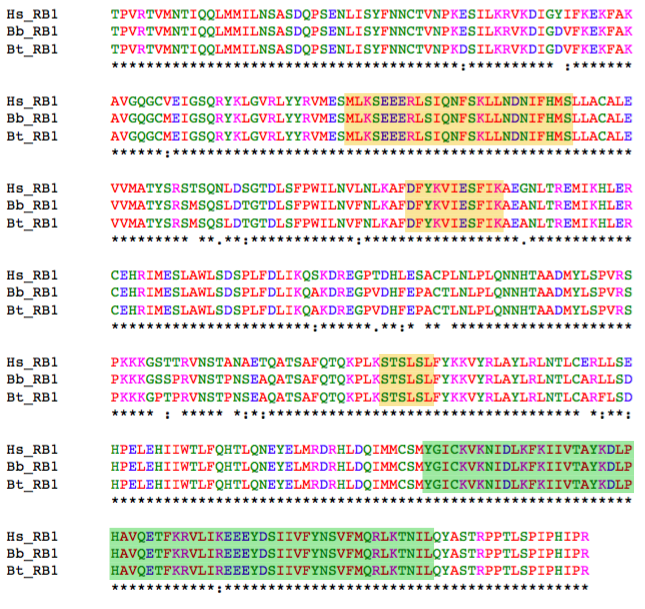


**Supplementary Figure 1. The structure of the Retinoblastoma-1 pocket domain is highly conserved between humans and bovids.** The sequences of *Homo sapiens* (Hs_RB1; GI:974999693), *Bubalus bubalis* (Bb_RB1; GI: 594080050), and *Bos taurus* (Bt_RB1; GI: 115304733) Retinoblastoma-1 ortholog pocket domains were aligned with CLUSTAL 1.2.1 and colored for residue types. Highlighted is the region containing residues for interaction of the RB pocket domain with the E2F transactivation domain (E2F-TA) (yellow) (31), and the region containing residues for the interaction of the RB pocket domain with the Human papillomavirus E7 LXCXE-containing protein (green) (32).

**
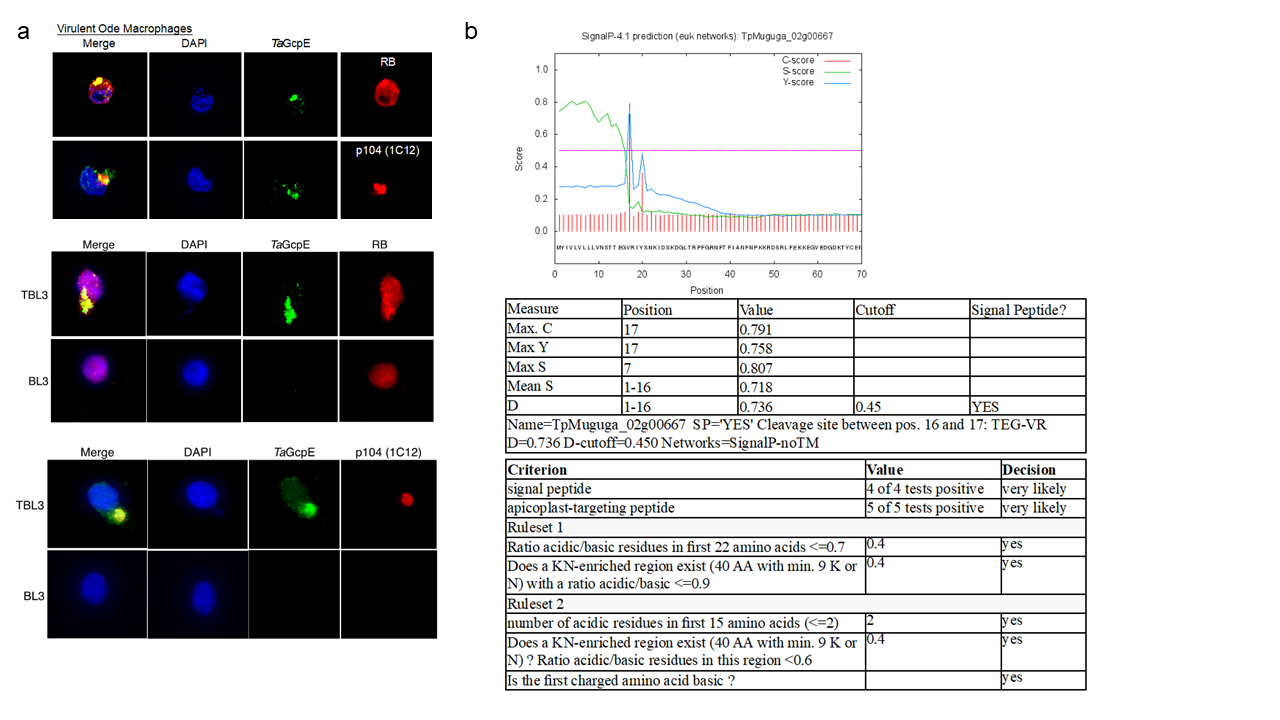
**

**Supplementary Figure 2. A polyclonal anti-peptide antibody and SignalP prediction support *Ta*GcpE as a possibly secreted protein and co-localizes with both the host protein Retinoblastoma-1 (RB) and the parasite (p104 clone 1C12) in virulent Ode macrophages and TBL3 cells.** (a) Immunofluorescence staining of the indicated host (RB) and parasite (P104) markers in *T. annulata* infected Ode macrophages (top) and uninfected BL3 as well as *T. annulata* infected TBL3 cells. The TpMuguga_02g00667 protein sequence was submitted to both (A) SignalP (http://www.cbs.dtu.dk/services/SignalP/) and (B) PlasmoAP (http://v4-6334.plasmodb.org/restricted/PlasmoAPcgi.shtml) with default settings. TpMuguga_02g00667 passed all criteria for predicted secretion and apicoplast signals, as indicated.

**Supplementary Tables**

**Supplementary Table 1.** Shown are the identifiers for hits for both RB-binding motifs, as well as the sequences for the motif hits and the peptides used to screen for functional RB-binding activity. Of the original list of 30 proteins, 15 were removed because the putative RB-binding domain was predicted to be on the cytoplasmic side of the parasite plasma membrane (TMHMM), or within a known functional domain in *T. parva*.

| **ELM ID [Regex]** | ***T. parva* protein** | ***T. parva* Motif** | ***T. annulata* protein** | ***T. annulata* Motif** | **Experimental Peptide** |
| --- | --- | --- | --- | --- | --- |
|  |  |  |  |  |  |
|  |  |  |  |  |  |
| ELME000007 [[LI].C.[DE]] | 01g00476* | LHCFD | TA20650 | LYCCD, LHCLD | LLHCFDSVK |
|  |  |  |  |  |  |
|  |  |  |  |  |  |
|  | 04g00024* | IECNE | TA17185 | IECDE | KIECNENHY |
|  | 01g00431 | IQCHE | TA20790 | IQCHE | FIQCHEKEF |
|  | 02g00667 | LKCKE | TA14455 | LKCKE | VLKCKELKK |
|  | 02g00758 | LTCRD | TA14945 | LSCRD | QLTCRDDSS |
|  | 03g00197 | LDCDE | TA03305 | ITCSD, LDCDE | TLDCDEASV |
|  | 03g00263 | ITCND | TA03615 | LECLE | LITCNDAHV |
|  | 04g00427 | LRCVD | TA08370 | LRCVD | QLRCVDNVF |
| ELME000301 [..[LIMV]..[LM][FY]D.] | 04g00896* | SVLVNLYDN, SVIANLYDI | TA11050 | SILVNLYDL, IIISNLFDI, SILVNLYDL, KVLSNLYDP | LDGKTSVLVNLYDNKELGP, IERQTSVIANLYDIERMGI |
|  |  |  |  |  |  |
|  | 01g00003 | KFLSILFDN | TA16050 | FKLFNLYDG, KCMNIMYDN, DNMFTLFDL | VTISSKFLSILFDNNTFKL |
|  | 01g00401 | DEIVNLYDS | TA20890 | DEIVNLYDS | IPKIVDEIVNLYDSCNTVE |
|  | 02g00019 | PGVFDLFDV | TA11485 | PGVFDLFDV | ESKGAPGVFDLFDVDITKN |
|  | 02g00134 | ESIKSLFDA | TA11540 | ESIKSLFDA | ATNNAESIKSLFDAGVDVF |
|  | 02g02355 | GVIQLLYDR | TA12035 | KEVYQLFDK, GVIQLLYDR | RDELQGVIQLLYDRYNKYI |
|  | 03g00786 | SQIQKLYDS | TA18220 | SEIQKLYDS | EKMDVSQIQKLYDSMIKSI |
